# Impact of workforce characteristics and monetary incentives on uptake of health and wellbeing initiatives in the United Kingdom

**DOI:** 10.1371/journal.pgph.0003984

**Published:** 2025-03-17

**Authors:** Adejoke Edet, Laura Kudrna, Laura Quinn

**Affiliations:** 1 Medicine and Occupational Health, EPCo Ltd, Fawley, United Kingdom; 2 Department of Applied Health Sciences, University of Birmingham, Birmingham, United Kingdom; Universiti Malaya, MALAYSIA

## Abstract

There are economic and social benefits associated with promoting the health and wellbeing (HWB) of workers. The workplace is an important setting for HWB promotion, however, small and medium size enterprises (SMEs) are less likely to offer these programmes. Uptake is also uneven across demographic groups, contributing to inequalities outcomes. This study investigates if uptake of HWB promotion programmes in SMEs differs by employee demographics and if these factors interact with the effectiveness of organisational-level monetary incentives to improve uptake. In a secondary analysis of quantitative data from a cluster-randomised controlled trial, multilevel logistic regression models were fitted to examine the relationship between the outcome (uptake of HWB initiatives) and employee demographics (age, gender, ethnicity, education level). Models included interactions between the trial arm (monetary incentive or control) and employee demographics. Results showed that employees in the incentive arm had similar uptake of HWB initiatives compared to the control (adjusted OR 1.11, CI 0.72, 1.70, *p* = 0.64). In tests of the demographic factors, employees 55+ years had 56% lower odds of uptake (CI 0.25, 0.76, *p* = 0.003) compared to employees aged 17 to 24 years and these results were robust to treating age as a continuous variable. There were no statistically significant interactions between the incentive and the other employee demographic groups for the uptake of HWB initiatives. Organisational-level monetary incentives do not appear to differ in their effectiveness according to employee demographics, although some sub-groups appeared under-represented in the trial including ethnic minorities and those with education below degree-level. Older workers in SMEs may be less likely to engage in workplace HWB initiatives and could be targeted in terms of workplace HWB implementation, policy, and research.

## Introduction

The economic and social impacts of poor health and wellbeing in workers are substantial. The World Health Organisation (WHO) estimates a negative economic impact of 4 to 6% of gross domestic product (GDP) for most countries [1]. In the UK, 141 million days were lost through sickness absence in 2018 [2] with an estimated cost of £18 billion in lost productivity which is predicted to rise to £26 billion by 2030 [3]. In addition to the direct costs of lost productivity, there are indirect costs, such as healthcare costs, that are greater [3]. The impact of poor health and wellbeing in workers is not limited to the workers and their workplaces. It also has an adverse impact on the health of their families, the community and wider society [4]. Consequently, interventions that improve the health and wellbeing (HWB) of workers will have a positive public health impact beyond the workplace.

Globally, there are an estimated 3.3 billion people in employment [5], and in the UK, there are 32.5 million people aged 16 years and over in employment [6], representing about half of the UK population. Economically active people spend, on average, a third of their time in the workplace [1]. The workplace is recognised as one of the priority settings for the promotion of health and wellbeing [7] and it offers opportunities to reach parts of the population that may not have access to health information in other settings. Furthermore, the workplace has established organisational structures and infrastructure that can facilitate the introduction of health and wellbeing initiatives [8]. The WHO considers workplace health programmes to be one of the ‘best-buy’ options for preventing and controlling non-communicable diseases (NCDs) and for boosting mental health [9]. Healthy workers are an essential prerequisite for economic development and productivity [10].

The promotion of the HWB of the workforce provides benefits to organisations, communities and the economy [11]. Some of the benefits include boosting of employee performance and productivity, improved staff retention and reduced sickness absence [11,12]. Workplace health promotion can be a useful strategy for addressing presenteeism [13], for preventing stress, and for creating positive working environments where employees and organisations thrive [11]. It can improve corporate image, employee morale and job satisfaction, and provide positive spillover effects to families and wider society [14].

Despite these benefits, the provision of workplace HWB promotion is variable. Large employers are more likely to offer HWB promotion programmes than smaller employers [15–17]. In the UK, small and medium-sized enterprises (SMEs) are companies with 10 to 250 employees [18], and they account for about 99% of businesses [19]. Globally, SMEs account for 90% of businesses and over half of employment [20]. The impact of the workplace as a setting for health promotion may be limited to the extent to which the workforce in SMEs can be reached [17]. There is evidence in the literature that one of the barriers to the provision of workplace HWB programmes in SMEs is the cost of implementing the programmes [21,22]. This suggests that offering financial incentives to SMEs could facilitate increased workplace HWB promotion.

It is, however, plausible that financial incentives impact some sub-groups of employees differently. Previous studies show that employee demographics are associated with uptake and participation in HWB activities [23,24], although the direction of influence is not consistent across studies. For instance, some authors report higher rates of participation in older workers while others report same in younger workers [23]. This is important because differential uptake may contribute to inequalities in outcomes.

The Thrive at Work incentives trial was the cluster randomised controlled trial (cRCT) from which the data for this study derives. The trial is believed to be the first cRCT to evaluate the effectiveness of an organisational-level monetary incentive for workplace HWB in SMEs. Results showed that the incentive motivated the employers in the intervention arm to introduce workplace HWB programmes by providing more information, activities, or services about mental health, musculoskeletal (MSK) health or lifestyle health [25]. However, organisational-level incentivisation showed no effect on employee uptake of the HWB initiatives [25]. This study investigates whether other factors, specifically employee demographics, affects uptake of HWB initiatives in SMEs, and if these factors interact with monetary incentives to promote uptake.

## Methods

### Ethical statement

This study was approved under University ethical regulations REGO-2018-2230 AM01. All participants provided written informed consent, which was checked verbally before the interviews began. Participants were able to withdraw from the study at any time and for any reason prior to publication. No special consent procedures were required for any groups such as minors or those with intellectual disabilities.

### Study design and population

A secondary analysis of quantitative data from the Thrive at Work cluster-randomised controlled trial (cRCT). The full trial methods are described in full elsewhere [25]. In brief, the trial included 100 SMEs representing a diverse range of industries in the West Midlands of the UK. The study was based on a random sample of employees from the participating SMEs. Data were collected from participants from 1 Nov 2018 – 31 Jan 2020.

### Inclusion criteria

**Cluster-level.** To be eligible, SMEs had to have between 10 and 250 employees; be receptive to implementing HWB initiatives in the workplace (with or without financial incentives); and be willing and able to provide organisational-level data, allow employees time to complete questionnaires, and allow senior executives to be interviewed [26].

**Individual-level.** Employees were eligible if they were 16 years or older and had an employment contract with the employer, were willing to participate and able to provide written consent [26].

### Intervention groups

The intervention was a monetary incentive provided via bank transfer to employers, which is described in full elsewhere [25]. There were two levels of the intervention. Those receiving the high level (Group 1) were eligible to receive up to £200 per employee, capped at a maximum of £10,000. Those receiving the lower level (Group 2) were eligible to receive up to £100 per employee, capped at maximum of £5,000. The intervention was also paid in two installments. The first transfer was not conditional on performance and paid according only to level of the incentive and number of staff. SMEs receiving the higher incentive level were paid £60 per employee and those receiving the lower level were paid £30 per employee. The second transfer was conditional on completing a self-assessment form with 43 criteria about the health and wellbeing initiatives provided and activities, such as support from management and information or activities related to mental health, musculoskeletal (MSK) health, and lifestyle health. Employers were able to tailor the HWB initiatives and activities they provided to suit their workforce.

### Control groups

There were two control groups. Both control groups received no monetary incentive. The single control group (Group 3) had baseline, midline, and endline assessments. The double control group (Group 4) had endline observations only to enable an examination of the effect of measurement (reactivity effects, see full discussion in original trial manuscript [25]).

### Data collection

Our study re-analysed quantitative data derived from questionnaire-based interviews (assessments) that were conducted using a random sample of employees from the participating SMEs. The questionnaire was tested for length and content in some of the SMEs that did not meet the inclusion criteria, and was revised, before being used in the trial [26]. Baseline assessments were conducted prior to the start of the intervention to avoid ascertainment bias and were carried out for three of the four trial arms. Baseline assessments were not conducted for one of the two control arms to assess measurement reactivity (see above). Post-intervention assessments were conducted at 12 months in all four arms. The researchers received training to ensure that the data were collected systematically. Standardised methods of data collection were used to ensure comparability and to minimise errors. Password-protected tablet devices were used for data collection [26].

### Outcome variable

The outcome of interest in this study was the employee’s self-reported uptake of the workplace HWB initiatives. A composite (binary) variable was generated based on the employee’s responses to three questions about whether they used or took part in any workplace HWB offer provided by their employer:

-  an initiative (information, activities and/or services) related to mental health
-  an initiative (information, activities and/or services) related to healthy lifestyle
-  an initiative (information, activities and/or services) related to MSK health

For this study, a positive response to one or more of the questions was considered a ‘Yes’ and a negative response to all the questions was considered a ‘No’.

### Independent variables

The independent variables for this study were the employee demographics: age, gender, ethnicity and educational level. Age was categorised: 17–24, 25–34, 35–44, 45–54 and ≥ 55 years, to enable the targeting of workplace HWB interventions at specific age-groups as indicated by the study findings. Gender categories were self-reported male or female. Ethnicity categories were White British/White Other, Mixed, Asian/Asian British, Black/African/ Caribbean/Black British and Other. Educational level was categorised as None/Other = No formal qualification/Other; Apprenticeship; Level 1 = 1–4 GCSEs or equivalent qualification; Level 2 = 5 GCSEs or equivalent qualification; Level 3 = 2 or more A-levels or equivalent qualification, and Level 4 = Bachelor’s degree or equivalent and higher qualifications.

### Statistical analysis

To increase power for interaction analyses and to simplify the presentation and interpretation of results, the two intervention arms from the original trial were combined into a single intervention group and the two control arms were combined into a single control group.

We used descriptive statistics to summarise the individual-level employee demographics (age, gender, ethnicity and educational level) for the intervention group and for the control group at baseline and endline.

For regression analyses, there were three key sets of analyses:

(1) Multi-level logistic models were fitted to examine the relationship between the outcome (uptake of HWB initiatives) and treatment arm.
(2) The models in (1) were adjusted to include employee demographics: age, gender, ethnicity and educational level.
(3) Interaction terms between the trial group and employee demographics were added to the model in (2) to test for any differential effects of the intervention (monetary incentive) by demographic group.

In all models, the the fixed effects were trial arm (intervention or control) and period (baseline or endline) and random effects were clusters of SMEs. Interactions were evaluated using the Wald tests. Odds ratios with 95% confidence intervals were calculated to examine the strength of the associations. Statistical significance (two-tailed *p* value) was set at <0.05. Stata v.15 was used for statistical analyses.

### Sample size and power

The power calculations for the original study used a Bayesian assurance analysis approach based on an 80% probability of observing a 95% posterior credible interval that excluded zero for dichotomous outcomes with a baseline of 10% to 50% [25,26]. It was estimated that 132 SMEs were needed to achieve this, however, the final sample only included 100/132 (75%) of SMEs because some SMEs decided not to participate after they were recruited. The implications of this are addressed in the Discussion.

## Results

A total of 750 employees (519 in the intervention group; 231 in the control group) completed baseline assessments. Table 1 presents the employees’ baseline characteristics. Employee demographics were broadly similar between the two groups. The number of employees in the intervention group was greater than double the number of employees in the control group. This was because baseline assessments were collected from only one of the two control arms in the original research to assess for measurement reactivity. The employees were predominantly White British/White Other. There were a range of industrial sectors in the sample: 18% of SMEs were Class A on the UK Standard Industrial Classification of Economics Activities (manual and secondary sector such as manufacturing and construction,), 42% were Class B (service and tertiary sector), and 40% were Class C (social and public sectors such as arts and schools) [25,26].

**Table 1:** Demographic characteristics of employees at baseline by trial arm.

		Total (N=750)
	Incentive group (n=519)	Control group (n=231)
**Age categories** n (%)		
17 - 24 years	78 (15.0)	41 (17.8)
25 - 34 years	164 (31.6)	86 (37.2)
35 - 44 years	116 (22.4)	46 (19.9)
45 - 54 years	104 (20.0)	34 (14.7)
≥ 55 years	57 (11.0)	24(10.4)
**Gender** n (%)		
Female	301 (58.0)	144 (62.0)
**Ethnicity** n (%)		
White British/White Other	425 (81.9)	197 (85.3)
Mixed	10 (1.9)	9 (3.9)
Asian/Asian British	52 (10.0)	14 (6.1)
Black/African/Caribbean/Black British	27 (5.2)	8 (3.4)
Other/no answer	5 (1.0)	3 (1.3)
**Educational level** n (%)		
None/Other	34 (6.5)	14 (6.1)
Level 1	27 (5.2)	8 (3.5)
Level 2	54 (10.4)	24 (10.4)
Level 3	100 (19.3)	71 (30.7)
Level 4	286 (55.1)	100 (43.3)
Apprenticeship	18 (3.5)	14 (6.1)

### Effect of the intervention on uptake of health and wellbeing initiatives

The employees in the intervention group had similar odds of uptake of health and wellbeing initiatives compared to employees in the control group (OR 1.07, CI 0.69, 1.64, *p* = 0.77).

### Employee demographics and uptake of health and wellbeing initiatives

Employees aged 55 years and older had 56% lower odds of uptake of HWB initiatives (adjusted OR 0.44, CI 0.25, 0.76) compared to employees aged 17 to 24 years, *p* = 0.003. The pattern of lower odds with increasing age held when age was treated as a continuous variable (adjusted OR 0.98, CI 0.97, 0.99, *p* = 0.001). There were no significant differences in uptake of HWB initiatives between any of the other age groups. In addition, the analysis did not show any statistically significant associations between the other employee demographics (gender, ethnicity and educational level) and uptake of workplace HWB initiative (Table 2).

**Table 2:** Associations between uptake of HWB initiatives and intervention and employee demographics at endline.

Uptake of HWB initiatives				
	Yes n (%)	No n (%)	Adjusted OR (95% CI)	*p*
**Trial group**				
Control	109 (33.3)	218 (66.7)	Ref	–
Intervention	174 (36.1)	308 (63.9)	1.11 (0.72, 1.70)	0.64
**Age group**				
17 - 24 years	38 (34.9)	71 (65.1)	Ref	–
25 - 34 years	101 (38.3)	163 (61.7)	0.99 (0.66, 1.48)	0.94
35 - 44 years	69 (37.9)	113 (62.1)	0.94 (0.61, 1.47)	0.80
45 - 54 years	52 (35.4)	95 (64.6)	0.73 (0.46, 1.17)	0.13
≥ 55 years	23 (21.5)	84 (78.5)	0.44 (0.25, 0.76)	**0.003**
**Gender**				
Male	183 (57.5)	135 (42.5)	Ref	–
Female	100 (20.4)	391 (79.6)	1.21 (0.92, 1.60)	0.17
**Ethnicity**				
White British/White Other	230 (34.7)	433 (65.3)	Ref	–
Mixed	6 (30.0)	14 (70.0)	1.03 (0.43, 2.44)	0.95
Asian/Asian British	31 (41.3)	44 (58.7)	0.77 (0.49, 1.21)	0.26
Black/African/Caribbean/Black British	14 (42.4)	19 (57.6)	1.07 (0.59, 1.94)	0.82
Other/no answer	2 (11.1)	16 (88.9)	0.37 (0.11, 1.20)	0.10
**Educational level**				
None/Other	14(29.2)	34 (70.8)	Ref	–
Level 1	6 (20.7)	23 (79.3)	0.68 (0.28, 1.66)	0.40
Level 2	27 (32.1)	57 (67.9)	1.01 (0.51, 2.00)	0.97
Level 3	54 (29.7)	128 (70.3)	0.85 (0.46, 1.58)	0.61
Level 4	175 (40.1)	261 (59.9)	1.23 (0.68, 2.22)	0.49
Apprenticeship	7 (23.3)	23 (76.7)	0.93 (0.39, 2.22)	0.87

### Interaction effects

Interactions between the intervention (incentive) and the demographics were examined to determine if the outcome differed for different employee demographic groups. These showed that there were no statistically significant interactions as presented in Table 3. Wald tests were completed, and these were not statistically significant at the 5% level (see footnote to Table 3).

**Table 3:** Association between intervention and uptake of HWB initiatives by demographic groups at endline.

Uptake of HWB initiative				
Intervention		Control		
	**no uptake/no subgroup (%)**	**no uptake/no subgroup (%)**	**Intervention effect OR (95% CI)** ^*^	***p***
**Age group**				
17 - 24 years	23/63 (36.5)	15/46 (32.6)	1.12 (0.51, 2.45)	0.78
25 - 34 years	64/158 (40.5)	37/106 (34.9)	1.06 (0.61, 1.87)	0.83
35 - 44 years	36/102 (35.3)	33/80 (41.3)	0.84 (0.44, 1.61)	0.60
45 - 54 years	36/100 (36.4)	16/48 (33.3)	1.28 (0.65, 2.53)	0.48
≥ 55 years	15/60 (25.0)	8/47 (17.0)	1.26 (0.51, 3.10)	0.61
**Gender**				
Male	70/192 (36.5)	30/125 (24.0)	1.28 (0.74, 2.21)	0.38
Female	104/289 (36.0)	79/202 (39.1)	0.96 (0.59, 1.55)	0.86
**Ethnicity**				
White British/White Other	143/385 (37.1)	87/278 (31.3)	1.11 (0.72, 1.77)	0.61
Mixed	3/12 (25.0)	3/8 (37.5)	0.96 (0.15, 6.13)	0.97
Asian/Asian British	17/49 (34.7)	14/26 (53.8)	1.28 (0.51, 3.20)	0.59
Black/African/Caribbean/Black British	10/24 (41.7)	4/9 (44.4)	0.44 (0.13, 1.48)	0.19
Other/no answer	1/12 (8.3)	1/6 (16.7)	0.30 (0.02, 3.98)	0.36
**Educational level**†				
None/Other	11/32 (34.4)	3/16 (18.8)	1.24 (0.39,3.95)	0.71
Level 1	4/21 (19.0)	2/8 (25)	0.71 (0.16, 3.17)	0.65
Level 2	19/56 (33.9)	8/28 (28.6)	1.04 (0.43, 2.50)	0.93
Level 3	33/111 (29.7)	21/71 (29.6)	0.84 (0.43, 1.62)	0.60
Level 4	102/250 (40.8)	73/186 (39.2)	1.25 (0.76, 2.04)	0.38
Apprenticeship	5/12 (41.7)	2/18 (11.1)	1.40 (0.29, 6.79)	0.67

* Wald tests - Age:Incentive *p* = 0.87, Gender:Incentive *p* = 0.29, Ethnicity:Incentive *p* = 0.47, Educational level:Incentive *p* = 0.86.

## Discussion

### Summary of the findings

We re-analyzed data from a workplace-based cRCT of 100 SMEs to look at inequalities in uptake of HWB initiatives. We determined whether employee demographics were associated with differential HWB uptake, and if there were any interactions between demographics and a monetary incentive that was randomly allocated to improve uptake. We found that employees aged 55 years or older had lower uptake of HWB initiatives in general, but that uptake of health and wellbeing initiatives was not affected by the interaction between the monetary incentive and demographic groups.

### Age

In this study, employees aged 55 years or older were less likely to take up workplace HWB initiatives. This is consistent with other studies that found that participants in workplace health promotion programmes (WHPPs), were younger [27,28], however, the HWB offer were predominantly physical fitness-based which may explain the greater interest and participation among younger employees. It is recognised that people over 50 years of age are more sedentary [29]. HWB offerings in the Thrive at Work study were diverse and tailored by the employer according to SME needs, however, we could not ascertain if there were more physical fitness-based offerings, which may have impacted participation in older employers.

This study’s findings differ from other studies [30–32] that report that older employees are more likely to participate in WHPPs. Nevertheless, direct comparison between studies is difficult as the studies generally dichotomized participant age, e.g., below 30 years and above 30 years, in contrast to the narrower age categories we used in this study to show more targeted differences. Consequently, given the rather crude dichotomization used in the studies, it is plausible that lower participation in the older employees was masked by higher participation in younger employees in the same broad category. However, given the multiple comparisons in this study, it is plausible that statistical significance could have arisen by chance.

### Gender

In contrast to many workplace HWB studies that report higher participation in females [23,24,27,33,34], this study did not identify a significant association between gender and uptake of HWB initiatives. However, the result tended in this direction. We did not analyse the specific types of HWB initiatives that employers provided or the specific activities that the employees engaged in. Furthermore, a composite measure was used for employee response to the question about uptake of HWB initiatives due to sample size considerations. It is possible that some SMEs did not offer HWB programmes that generate more interest in women, such as weight management and healthy nutrition programmes [35,36], negatively affecting uptake.

### Ethnicity

Employees in our study were predominantly White British/White Other, reflecting the wider West Midlands and UK population estimates [37,38]. Future research needs to include more diverse samples to better understand inequalities. Ethnicity is not reported in many studies on employee participation in workplace health promotion programmes [23,39] This study did not demonstrate a significant association between the employee’s ethnicity and uptake of workplace HWB initiatives, which is consistent with findings in the review by Robroek et al [23].

Contrastingly, a US study conducted within a single workplace found that White participants were more likely to enroll in a WHPP than Non-Whites [40]; however, the outcome measure was the employee’s indication of *intention* to participate rather than their actual participation making direct comparison with this study challenging.

### Education

There is an educational gradient to health-seeking behaviour [41,42], however, this was not demonstrated in this study. This is in contrast to previous studies that found that employees with higher educational level were more likely to participate in WHPPs [16,31]. In this study, there was no significant difference in uptake of HWB initiatives between employees of different educational levels. Of note, employees in this study were relatively well educated with about 75% having 2 or more A-levels or higher educational qualifications. Consequently, it is possible that the employees already maintained healthy lifestyles in their day-to-day life and did not consider it necessary to utilise or engage in the workplace offer. Again, more diverse trial samples are needed for tests of inequalities.

### Interaction of monetary incentive and employee demographics

In this study, there was no significant interaction between the incentive intervention and employee demographics for the outcome. Several studies [43,44] have investigated the effectiveness of individual level financial incentives in motivating employee participation in WHPPs, however, our study is the first to examine the interaction between an organisational-level financial incentive and employee uptake of HWB initiatives. Our results shows that differences in the intervention effect did not substantively differ between demographic subgroups, although some subgroups (education, ethnicity) were not well sampled.

### Strengths and limitations

A strength of this study is its focus on inequalities in the uptake of health and wellbeing initiatives, which can contribute to reducing disparities and inequities in health and wellbeing by informing populations to target. This an important addition to the first published analyses of the original trial, which did not look at inequalities in uptake [25]. The challenges of conducting workplace-based randomised trials are recognised and it is, therefore, prudent to make full use of existing data [45]. Our study was based in the West Midlands, nevertheless, some of the participant demographic characteristics such as age and ethnicity were reflective of the wider population, increasing the generalisability of the study findings.

One limitation of this study is that post-hoc power computations were not conducted as part of the original trial. Whilst the analysis in this study relates to one of the secondary outcomes of the original trial, the sample size was calculated on the basis of the primary outcome. Therefore, this secondary analysis of the trial data may have been underpowered to detect differences in uptake of HWB initiatives in the different groups. The original study did not meet its target sample size of 132 SMEs and there was self-selection among the 100 SMEs who did participate, which could bias the results towards SMEs who are most interested or motivated to take part in research and HWB initiatives. Furthermore, the small sample sizes within some of the demographic groups may have obscured true moderate-sized differences.

### Implications of the results

This study suggests that older workers in SMEs are less likely to engage in workplace HWB initiatives. From a public health perspective, life expectancy is increasing and the workforce is aging. It is estimated that the proportion of workers in Europe aged 55 to 64 years will increase by one-third come 2060 relative to the 2013 rate. It is, therefore, important from both policy and implementation perspectives to ensure that this expanding population is not disadvantaged with respect to relevant opportunities to maintain and improve their health and wellbeing.

The study findings also suggest that offering organisational-level monetary incentives does not lead to inequalities in uptake of workplace health and wellbeing initiatives with respect to employee age, gender, ethnicity and educational level. However, given the post-hoc nature of the analysis and the small subgroup sizes, these results deserve further investigation in more diverse samples. Future research on demographic differences in HWB should be powered to detect demographic differences in HWB uptake where resources allow.

This study analysed data derived from a cluster randomised trial that used a repeated cross-sectional sampling approach. Future research using a longitudinal design following the same employees over time may enable the assessment of behaviour change in the same workers with more accurate determination of the effect of the HWB intervention.

SME workers remain an important target group with respect to improving the health and wellbeing of the population and older workers are an important focus for future research to facilitate greater understanding of their HWB needs and the workplace-based interventions that are effective for this population.

In conclusion, this secondary analysis of cRCT data found lower participation in workplace HWB initiatives in older SME employees, however, there were no differences in HWB uptake in relation to the gender, ethnicity and educational attainment of the employees. Furthermore, there were no differences in the effect of the incentive within the different demographic groups. The workplace remains an important area for health and wellbeing promotion, further research to reduce age-related inequalities in relation to workplace HWB is required.
